# Gut Glucosinolate Metabolism and Isothiocyanate Production

**DOI:** 10.1002/mnfr.201700991

**Published:** 2018-07-05

**Authors:** Arjan Narbad, John Trevor Rossiter

**Affiliations:** ^1^ Quadram Institute Bioscience, Food Innovation and Health ISP Norwich Research Park Norwich Norfolk NR4 7UA UK; ^2^ Department of Life Sciences, Imperial College London SW7 2AZ UK

**Keywords:** glucosinolates, gut bacteria, isothiocyanates, myrosinase, nitriles

## Abstract

The glucosinolate‐myrosinase system in plants has been well studied over the years while relatively little research has been undertaken on the bacterial metabolism of glucosinolates. The products of myrosinase‐based glucosinolate hydrolysis in the human gut are important to health, particularly the isothiocyanates, as they are shown to have anticancer properties as well as other beneficial roles in human health. This review is concerned with the bacterial metabolism of glucosinolates but is not restricted to the human gut. Isothiocyanate production and nitrile formation are discussed together with the mechanisms of the formation of these compounds. Side chain modification of the methylsulfinylalkyl glucosinolates is reviewed and the implications for bioactivity of the resultant products are also discussed.

## 1 Introduction

During the cooking process of cruciferous vegetables, myrosinase activity and associated protein specifier proteins are usually destroyed unless strict cooking times are adhered to.^1^ Despite the thermal destruction of plant myrosinase activity the intake of cooked *Brassica* vegetables still results in the formation of bioactive isothiocyanates (ITCs) and nitriles which arise from the metabolism of glucosinolates (GSLs) by the human gut microbiota. While there is a great deal of information concerning the beneficial effects of GSLs hydrolysis products on human health^2^, ^3^, ^4^, ^5^, ^6^ very little is known about the importance of the gut microbiota in generating these bioactive GSL products. Generally, there has been little research into the metabolism of GSLs by bacteria particularly those of human origin. The lack of intensity of research into this area of GSLs is surprising as the human gut microbiota acts as a gateway for the formation of these key anticancer metabolites. In order to review the topic, bacteria of extraintestinal origin are also discussed to generate a wider picture of bacterial myrosinases. **Table** 1 shows the work in relation to animal and intestinal models while **Table** 2 details the work that has been carried out with pure bacterial cultures and includes the GSL substrates and their identified hydrolysis products.

**Table 1:** In vivo and in vitro fermentations

	Analysis						
	DS‐GSL	GSL	ITC	NIT	OP	% conversion of GSL/DS‐GSL (time)	Ref.
Rat‐diet supplemented with *B. thetaiotaomicron*		1	9	ND		100 (36 h)	19
Rat cecal microbiota		4	ND	trace		100 (24 h)	20
Cecal microbiota with MRS media		4	15	trace		39 (24 h)	
Human fecal inoculum		1	ND	ND	23	100 (30 h)	47
		2	ND	ND	24	100 (30 h)	
Human in vitro intestinal model		1	9	ND		100 (12 h)	90
Human in vitro intestinal model		4	15	16		60 (24 h)	67
		5*	19	20		(based on combined concentration of GSL 4 & 5)	
Rat intestinal microbiota		1	9	10		69 (6 h)	38
	1		9 (trace)	10	22		
*Lactobacillus* (LEM220) Rats		RSM	NT	NT		NA	15
*Escherichia coli* (EM0)		RSM	NT	NT		NA	17
*Bacteroides vulgatus* (BV8H1)		RSM	NT	NT		NA	

GSLs used as substrates; 1, sinigrin; 2, glucotrapaoelin; 4, glucoraphanin; 5, glucoerucin (^*^from in vitro interconversion of glucoraphanin to glucoerucin). Products: 9, allylisothiocyanate; 10, allylnitrile; 15, sulforaphane; 16, sulforaphane nitrile; 19, erucin; 20, erucin nitrile; 22, 3,4‐epithiobutanenitrile (3,4‐epithiobutanenitrile); 23, allylamine; 24, benzylamine. NA, not available; ND, not detected; NT, not tested; OP, other product; NIT = nitrile; DS‐GSL = desulfoglucosinolate, RSM = rape‐seed meal.

**Table 2:** In vitro assessment of pure bacterial strains shown to metabolize GSLs

		Fermentation				Cell‐free protein extract			
Bacterial species	Gram +/−	GSL	ITC	NIT	% GSL conversion 24 h (unless specified)	GSL	ITC Myr+, Myr−	NIT	Ref.
*Citrobacter* WYE1	−	1	ND	ND	100	1,2,3,4,5	9,12,14,15,19 Myr+	ND	34
*Bacillus* (isolates)	+	1	NT	NT	74/91/62/56	NT			21
*Pseudomonas*	−	1	NT	NT	NA	NT			21
*Lactobacillus*	+	1	NT	NT	NA	NT			21
*Lactobacillus* (LEM)	+	1	NT	NT	13–28 (5 d)	NT			15
		8	NT	NT	13–20 (5 d)	NT			15
*Streptomyces* (isolates)	+	1	NT	NT	43–67	NT			21
*Staphylococcus*	+	1	NT	NT	77	NT			21
*Lactobacillus agilis* R16	+	1	9	10	100	1	Myr−		22, 24
		5	19	20	100	NT			23
		6	ND	ND	11	NT			23
		4	ND	ND	10	NT			23
		2	12	11	90	NT			24
		3	14	ND	95	NT			24
*Enterococcus casseliflavus* CP1	+	1	9	10	100	1	Myr−		24
		5	19	20	100	NT			23
		6	Trace	ND	41	NT			23
		4	ND	Trace	53	NT			23
		2	12	11	90	NT			24
		3	14	13	100	NT			24
*Escherichia coli* VL8	−	1	9	10	90	1	Myr−		24
		5	19	20	100	NT			23
		6	17	18	87	NT			23
		4	15	16	91	NT			23
		2	12	11	100	NT			24
		3	14	13	100	NT			24
*Lactobacillus plantarum KW30*	+	4,6	ND	16,18,20	30–33	NT			25
*Lactococcus lactis* subsp. *lactis* KF147	+	4,6	ND	16,18,20	30–33	NT			25
*Escherichia coli* Nissle 1917	−	4,6	ND	16,18	65–78	NT			25
*Enterobacter cloacae*	−	1	NT	NT	100 (24–48 h)	1	Myr+		33
*Enterobacter cloacae* KS50	−	1	NT	NT	NA	1	Myr+		31
*Bacillus cereus* 10X	+	RSM	21	NT	NA	NT			91
*Bacillus cereus* St3									
*Lactobacillus gasseri*	+	4	ND	16	36–49	NT			26
*Lactobacillus acidophilus*	+	4	ND	16	36–49	NT			26
*Lactobacillus casei*	+	4	ND	16	36–49	NT			26
*Lactobacillus plantarum*	+	4	ND	16	36–49	NT			26
*Bifidobacterium pseudocatenulatum*	+	1,2	NT	NT	73–83 (48 h) for all strains for GSL (1). 84 (48 h) for *B. adolescents* with GSL (2)	NT			27
*Bifidobacterium adolescents*	+	1	ND	10			9		27
*Bifidobacterium adolescents*		2	ND	11		NT			27
*Bifidobacterium longum*	+	1,2	ND	NT		NT			27
*Bacteroides thetaiotaonicron* (II8)	−	1	9	ND	100 (36 h)	NT			19
*Escherichia coli* (various strains)	−	8	21	NT	3–26 (48 h)	NT			14
*Paracolobactrum aerogenoides*	−	8	21	NT	24–81 (48 h)	8	21	NT	14
*Aerobacter aerogenes*	+	8	21	NT	26–28 (48 h)	NT			14
*Bacillus subtilis*	+	8	21	NT	59–72 (48 h)	NT			14
*Staphylococcus epidermis*	+	8	21	NT	19 (48 h)	NT			14
*Proteus vulgaris*	−	8	21	NT	42–48 (48 h)	NT			14
*Escherichia coli* 0157:H7	−	1	9	NT	12 (5 d)	NT			30
*Lactobacillus curvatus* (various strains)	+	7	NT	NT	2.4–5.4 (6 d)	NT			28
*Lactobacillus plantarum* (various strains)	+	7	NT	NT	0.6–4 (6 d)	NT			28
*Pediococcus pentosaceus* (various strains)	−	7	NT	NT	5.02–11.3 (6 d)	NT			28
*Staphylcoccus carnosus* (various strains)	+	7	NT	NT	6.06–10 (6 d)	NT			28
*Pediococcus acidilactici*	+	7	NT	NT	2.92–3.16 (6 d)	NT			28
*Pediococcus pentosaceus*	+	1	9	NT	11.99 (12 d)	NT			29
*Escherichia coli* 0157:H7	−	1	9	NT	38.96 (12 d)	NT			29
*Listeria monocytogenes*	+	1	9	NT	19.04 (8 d)	NT			29
*Escherichia fecalis*	+	1	9	NT	9.05 (12 d)	NT			29
*Staphylococcus aureus*	+	1	9	NT	20.39 (8 d)	NT			29
*Staphylococcus carnosus*	+	1	9	NT	21.2 (8 d)	NT			29
*Salmonella typhimurium*	−	1	9	NT	28.02 (12 d)	NT			29
*Pseudomonas fluorescens*	−	1	9	NT	7.17 (12 d)	NT			29
*Listeria monocytogenes*	+	1	9	NT	53.2 (21 d, 21 °C)	NT			92
*Salmonella*	−	1	9	NT	59.9 (21 d, 21 °C)	NT			92

The presence or absence of the typical products, that is, ITC and/or nitrile, are indicated. % GSL conversion is given as the least to the maximum value for any number of isolates. NA = not available; ND, not detectable; NT, not tested. Myr+, myrosinase activity; Myr−, no myrosinase activity; NIT = nitrile. GSLs used as substrates: 1, sinigrin; 2, glucotropaeolin; 3, gluconasturtiin; 4, glucoraphanin; 5, glucoerucin; 6, glucoiberin; 7, sinalbin; 8, progoitrin. Products: 9, allylisothiocyanate; 10, allylnitrile; 11, benzylnitrile; 12, benzylisothiocyanate; 13, phenethyl nitrile; 14, phenethylisothiocyanate; 15, sulforaphane; 16, sulforaphane nitrile; 17, iberverin; 18, iberverin nitrile; 19, erucin; 20, erucin nitrile; 21, goitrin. RSM, rape seed meal GSL extract products, that is, ITC and/or nitrile, are indicated.

### 1.1 Isothiocyanate Production

**Figure 1:**
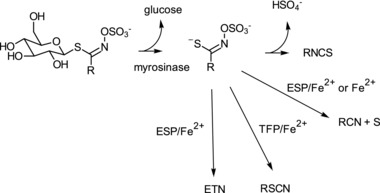
Generalized scheme of the hydrolysis of GSLs by plant myrosinases. RNCS, isothiocyanate; RCN, nitrile; RSCN, thiocyanate; ETN, epithionitriles; ESP, epthiospecifier protein; TFP, thiocyanate forming protein.

In general, the natural origins of ITCs are from the myrosinase catalyzed hydrolysis (**Figure** 1) of GSLs.^7^ Marine organisms are also known to produce ITCs such as the diterpenoids 10‐*epi*‐kalihinol I and 5,10‐bisisothiocyanato kalihinol G which have been shown to be biologically active.^8^ The functional activity of the ITCs resides in the electrophilic nature of the carbon atom of the N=C=S group which is able to undergo addition reactions with various nucleophiles.^9^, ^10^ With amines, thioureas are formed while with sulfhydryl groups dithiocarbamates are the products.^9^, ^10^ Since the diet is complex with a myriad of small molecules it is likely that ITCs react with many nucleophiles and not just with amines or sulfhydryls. The metabolism of ITCs in animal and human cells is via the glutathione (GSH) pathway and is reviewed by other authors in this special edition. To maximize the benefits of ITCs, it is of importance to understand how the human gut microbiota metabolizes GSLs to ITCs and to what extent these ITCs are further metabolized to form other products that may be more or less bioactive. The gut bacteria play a key role in generating ITCs but these are not always the only end products. Various microbiological studies examining GSL metabolism have been carried out using animals with modified diets and specific microbiotas as well as in vitro model fermentation systems inoculated with fecal or cecal bacteria (Table 1). The biotransformation studies with isolated individual bacterial cultures are listed in Table 2. The formation of goiter is a known phenomenon associated with a high intake of cruciferous vegetables in farm animals and humans.^11^ One of the first goitrogens to be discovered was 5‐ethenyl‐1,3‐oxazolidine‐2‐thione which is derived from 2(*R*)‐hydroxy‐3‐butenylglucosinolate (progoitrin)^12^ and was given the name goitrin.^13^ Eventually the link between bacterial GSL metabolism and production of goitrin from progoitrin was established.^14^ In this study, various fecal isolates were tested against progoitrin (Table 2) and *Paracolobactrum aerogenoides* was found to be the most active degrader. The myrosinase activity was also demonstrated in its cell‐free protein extract. Further evidence for the involvement of bacteria in GSL metabolism came from work with a *Lactobacillus* strain LEM220 which was able to degrade GSLs^15^ (Table 1). Rats fed on a GSL‐rich diet with a *Lactobacillus* LEM220 supplement developed goiter in comparison to controls which also confirmed the authors’ previous work.^16^ Further investigations revealed that gnotobiotic rats associated with *Escherichia coli* EM0 or *Bacteroides vulgatus* BV8H1 fed on a rape‐seed meal diet developed goiter, thus implicating activity of GSL metabolizing bacteria.^17^ As part of a screen for GSL metabolizing bacteria from human intestinal microbiota, *Bacteroides thetaiotaomicron* was isolated and found to convert sinigrin to allylisothiocyanate.^18^ This isolate was tested in gnotobiotic rats supplemented with sinigrin and it was found that allylisothiocyanate was produced in the digestive system, thus for the first time linking GSL metabolism with a specific bacterium.^19^

Other workers have investigated rat cecal microbiota with a combination of glucoraphanin and bacterial growth media.^20^ Here it was found that the rat cecal microbiota produced sulforaphane from GSL but only when supplemented with MRS (de Man, Rogosa, Sharpe), the media that supports the growth of *Lactobacilli*. Pretreatment of the rats with glucoraphanin prior to obtaining the cecal contents increased the ex vivo conversion of GSL to ITC suggesting induction of bacterial myrosinase activity.

Brabban and Edwards^21^ carried out an extensive study testing some 192 laboratory strains for their ability to metabolize sinigrin. All bacteria that degraded sinigrin in this study were Gram‐positive and included members of *Streptomyces*, *Bacillus*, and *Staphylococcus* derived from different sources (river Mersey, contaminated soil, and mushroom compost); however, the products of GLS metabolism by these bacteria were not identified. One of the most well‐studied bacterial strains of human gut origin is the Gram‐positive *Lactobacillus agilis* R16 isolated by Palop et al.^22^ which produces allylisothiocyanate from sinigrin. The authors could demonstrate myrosinase activity with intact cells but not with cell‐free protein extracts. Subsequent studies^23^, ^24^ with *L. agilis* R16 showed similar results with sinigrin except that allylnitrile was also a product. This study was expanded to include glucotropaeolin, gluconasturtiin, glucoraphanin, glucoerucin, and glucoiberin. The activity of *L. agilis* R16 with these GSL substrates showed a degree of substrate specificity as neither glucoraphanin nor glucoiberin was metabolized while gluconasturtiin produced only an ITC. Glucoerucin‐ and glucotropaoelin‐like sinigrin were converted to ITCs and nitriles.^23^, ^24^ It was not possible to demonstrate myrosinase activity with cell‐free protein extracts. Further studies were carried out with two bacterial strains *Enterococcus casseliflavus* CP1 and *Escherichia coli* VL8 that originated from human fecal material.^23^, ^24^ *E. coli* VL8 was able to metabolize all the GSLs tested (Table 2) to both nitriles and ITCs while *E. casseliflavus* CP1 was able to metabolize all GSLs tested to nitriles and ITCs with the exception of glucoraphanin and glucoiberin where only trace amounts of ITCs and nitriles were observed. Thus, it appears that the nature of the GSL side is an important factor in GSL metabolism. As with *L. agilis* R16, all attempts to identify myrosinase activity in vitro in *E. casseliflavus* CP1 and *E. coli* VL8 were unsuccessful. Mullaney et al.^25^ carried out a study comparing lactic acid bacteria with *Enterobacteriaceae* and in all cases the products of GSL metabolism were nitriles and not ITCs. In this study, glucoraphanin and glucoiberin were used and it was found that methylsulfinyl group of the side chain underwent reduction to the methylthio form and this is discussed in more detail later. A study by Lai et al.^26^ investigated the hydrolysis of glucoraphanin by various *Lactobacilli* in culture media and in all cases the corresponding nitrile was the major metabolic product. Three *Bifidobacteria* strains (*Bifidobacterium pseudocatenulatum*, *Bifidobacterium adolescentis, Bifidobacterium longum*) were examined^27^ for their ability to biotransform GSLs. All three strains were able to metabolize sinigrin during fermentation while *B. adolescentis* also tested positive for glucotropaeolin metabolism. In the case of *B. adolescentis*, the products of fermentation were allylnitrile and benzylnitrile while there is less information on the products from *B. pseudocatenulatum* and *B. longum*. The authors carried out further work examining a cell‐free protein extract from *B. adolescentis* and found myrosinase activity with the formation of allylisothiocyanate. Activation by ascorbate was marginal in comparison to plant myrosinases.^7^ Attempts to repeat this work with these *Bifidobacteria* strains (RIKEN, Japan Collection of Microorganisms) were not successful (unpublished data) and may indicate that this trait is either unstable over a period of time or requires an unknown trigger that induces the biosynthesis of the myrosinases in these bacteria.

Luciano et al.^28^ screened a number of bacteria for their ability to degrade sinalbin and found various strains including *Lactobacillus curvatus*, *Lactobacillus plantarum*, *Pediococcus pentosaceus, Staphylococcus carnosus, Staphylococcus aureus*, and *E. coli* 0157:H7 to be degraders with the latter being the most active. Further studies involved screening a variety of bacteria including *E. coli* 0157:H7 with sinigrin as a substrate with all strains producing allylisothiocyanate.^29^ More recent work with the GSL metabolizing *E. coli* 0157:H7 identified genes *bglA* and *ascbB* encoding 6‐phospho‐β‐glucosidases.^30^ Following gene disruption, the sinigrin degrading ability of this organism was substantially reduced. In order to confirm the functional role of these two genes, it would be desirable to complement or overexpress these enzymes in the deletion strains.

Recently, an isolate from the *Brassica* microbiome has been identified as *Enterobacter cloacae* KS50 and was shown to have myrosinase activity in cell‐free protein extracts.^31^ The first bacterial myrosinase purification was carried out by Tani et al.^32^ from *E. cloacae* 506.^33^ The myrosinase was purified to homogeneity by classical chromatography techniques with a molecular weight of 61 kDa. Since this early study only one other bacterial myrosinase has been purified from *Citrobacter* Wye1^34^ which has a molecular weight of 66 kDa and was shown to belong to the glycoside hydrolase family 3 (GH3) β‐*O*‐glucosidases. Cell‐free protein extracts produced ITCs, although during fermentation another product was detected but its identity was not established. The *Citrobacter* Wye1 myrosinase has been cloned and successfully expressed in *E.coli* and was shown to be a fully functional enzyme.^35^

### 1.2 Nitrile Formation

**Figure 2:**
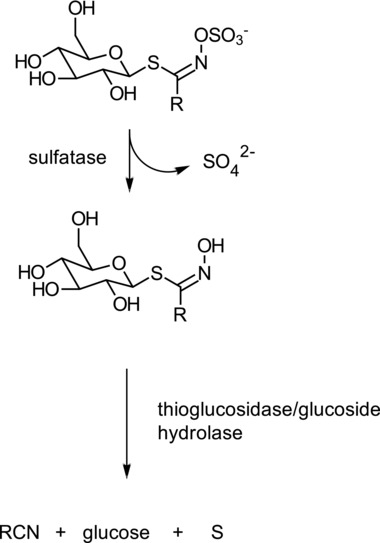
Hypothesized route to synthesis of nitriles (RCN) via desulfoglucosinolates (DS‐GSLs).

Cruciferous plants can possess specifier proteins namely epithiospecifier protein (ESP), thiocyanate forming protein (TFP), and nitrile specifier protein (NSP) which direct the myrosinase catalyzed hydrolysis of GSLs to nitriles, epithionitriles, and thiocyanates^36^ (Figure 1). As part of investigations into the mechanism of these specifier proteins it has been established that ferrous ions play a key role within the active site of the protein during catalysis.^37^ While nitriles are readily observed during the hydrolysis of GSLs in fermentation, so far no bacterial specifier proteins have been found or investigated that promote the formation of nitriles. Interestingly, an epithionitrile (ETN) has been observed in only one study^38^ (Table 1). In this particular case the substrate was not an intact GSL but DS‐sinigrin. As far as we are aware, TFP, NSP, and ESP‐like proteins have not been investigated for their ability to modify the products of GSLs or desulfoglucosinolates (DS‐GSLs) in bacterial systems. Sulfatases have been identified in many bacteria while few have been cloned and characterized.^39^, ^40^ A detailed study examining the metabolism of five DS‐GSLs in bacterial fermentations has recently been reported^24^ where it was shown that specific strains of *E. coli* VL8*, L. agilis* R16, and *E. casseliflavus* CP1 can utilize DS‐GSLs as a carbon source and produce nitriles. *L. agilis* R16 and *E. casseliflavus* CP1 however, could not metabolize DS‐glucoraphanin while the former was also unable to utilize DS‐gluconasturtiin. *E. coli* VL8 could metabolize all DS‐GSLs tested to their nitrile derivatives. Another study has shown that a recombinant β‐*O*‐glucosidase from *Caldocellum saccharolyticum* was able to transform a number of DS‐GSLs to their corresponding nitriles in the absence of ferrous ions.^41^ This suggests that the origin of nitriles during the fermentation of GSLs may well be a result of desulfation followed by hydrolysis (**Figure** 2). It is known that plant myrosinases can direct hydrolysis toward nitriles in the presence of ferrous ions without a requirement for a specifier protein.^42^, ^43^ A study examining GSLs incubated with the resting cells of *E. coli* VL8 indicated that the presence of ferrous ions shifted hydrolysis away from ITCs toward nitriles^24^ suggesting a ferrous ion dependency.

Other work has also shown that a recombinant β‐*O*‐glucosidase (bgl4) was able to hydrolyze DS‐gluconasturtiin to phenethylnitrile without the presence of ferrous ions and on the contrary these ions if present inhibited the hydrolysis.^44^ The generation of nitriles from DS‐GSLs following hydrolysis by a β‐*O*‐glucosidase is likely due to the spontaneous decomposition of the thiohyroxamic acid without a need for ferrous ions.^45^, ^46^ In order to understand nitrile production during GSL hydrolysis further detailed work is required, particularly the role of ferrous or other metal ion species. In this respect, the composition of fermentation media is of importance as the presence of metal ions here can potentially influence the outcome of GSL hydrolysis. Also the observation of an ETN in one study with DS‐sinigrin^38^ requires follow‐up as the presence of ferrous ions are unlikely to be the only factor in the generation of this nitrile derivative. The fact that amines can be produced during fermentations suggests the presence of bacterial nitrile reductases.^47^ These are a relatively new class of enzymes and one has been recently cloned and expressed from *E. coli* K‐12 and shown to reduce 7‐cyano‐7‐deazaguanine to an amine 7‐amino‐methyl‐7‐deazaguanine but has limited substrate specificity.^48^ Whether or not bacterial nitrile reductases exist for GSL‐derived nitriles remains an open question. Nitrilases are well known^49^ and it is possible that GSL‐derived nitriles are further hydrolyzed to carboxylic acids thus underestimating the prevalence of nitrile production. GSLs give rise to sulfate and in the presence of ferrous ions will also generate sulfur (Figure 1). In the human gut, the sulfate released by GSL hydrolysis is likely to be reduced to hydrogen sulfide by sulfate‐reducing bacteria.^50^ Hydrogen sulfide at high levels can have negative implications for human health and the importance of diet and microbiota in this respect is poorly understood.

### 1.3 Isothiocyanate Stability

As mentioned earlier, the outcome of the bacterial metabolism of GSLs may be influenced by the constituents of the growth media. It is quite possible that metal ions present in media may affect the nature of the products formed, for example, ferrous ions may shift GSL hydrolysis toward nitriles. Another factor likely to be of importance is the stability of the ITCs in the growth media. Various growth media are utilized depending on the bacteria in question and it is possible that ITCs can react with some of the media components as well as with components of the cell. Previous work has shown ITCs to be unstable in buffers and water^51^, ^52^ while others have determined the half‐lives of ITC conjugates^53^ which is clearly an important factor to consider when carrying out quantitative determinations of hydrolytic products.

### 1.4 The Analysis of Glucosinolate Metabolites

**Figure 3:**
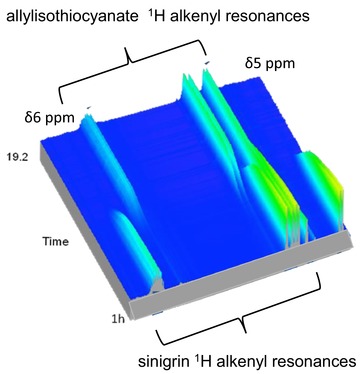
The metabolism of sinigrin monitored by ^1^H‐NMR over 19 h showing the changes in the proton resonances of the alkenyl region of sinigrin and allylisothiocyanate during metabolism. ^1^H‐NMR was carried out on a Bruker Avance DRX600 spectrometer, 14.1 T magnet, and 600 MHz proton resonance frequency.

Methods for the analysis of GSLs and their hydrolysis products are well reviewed^54^ and here we highlight some of the main problems concerning the measurement of GSL metabolites in the gut and fermentation models. During the consumption of *Brassica* vegetables not all of the products of GSLs hydrolysis can be accounted for and ideally the yield of ITCs from an intake of *Brassica* vegetables would be 100%, thus enabling the full potential of these health promoting compounds.^55^ In this respect, the method of analyzing ITCs is important as traditional methods such as GC‐MS and LC‐MS are likely to underestimate ITC concentrations if significant amounts are bound to protein via lysine and cysteine residues. Alternative methodology, that measures total ITCs, such as those that utilise the cyclocondensation reaction have been successfully used in a study examining rats fed with broccoli powder.^56^ This was in contrast to LC‐MS/DAD and GC‐MS analyses where no ITCs were detected. Treatment of the samples with excess GSH however, enabled the ITCs to be observed as their GSH conjugates on the basis that an excess of GSH displaces the ITC from the protein‐bound conjugates. Methodology has been developed for examining the protein adducts of 1‐methoxy‐3‐indolylmethyl glucosinolate (neoglucobrassicin) metabolites in various organs of mice and this is also potentially a valuable tool in the quantification of ITC protein adducts.^57^ The use of isotopically labelled GSLs in studying the metabolism of these compounds in animal models has been very limited. Studies have been carried out with radiolabelled ITCs and 3,4‐epithiobutanenitrile where these compounds were fed to rats and their disposition and pharmokinetics were determined.^58^, ^59^, ^60^ While recent advances in LC‐MS enables much of the metabolism of GSLs to be followed, there is still merit in using both radiolabelled and stable isotopically labelled GSLs. The use of radiolabelled ITCs has given important information on the distribution and pharmokinetics of these compounds but does not represent the true picture of GSL metabolism, particularly in terms of other products such as nitriles. ^1^H NMR has been successfully used to monitor GSL metabolism in a human fecal inoculum during an in vitro fermentation^47^ and identified two amine products. **Figure** 3 shows the metabolism of sinigrin by *L. agilis* R16 to give predominantly allylisothiocyanate in real time (unpublished data) using ^1^H‐ NMR. In vivo NMR is a powerful tool^61^ to study metabolism, yet has been little used in GSL research. With the known synthesis of [10‐^13^C,11,12‐^2^H_5_]glucoraphanin^62^ it is surprising that this GSL has not been utilized in metabolism work with humans or animal models where there could be scope for in vivo NMR spectroscopy. Other radiolabelled GSLs and stable isotopically labelled GSLs have also been synthesized which also would be useful in GSL metabolism studies.^63^, ^64^

### 1.5 Side Chain Modification

The range of GSLs tested during fermentations as well as the products formed are shown in Table 1. The most commonly examined GSL is sinigrin, mostly because of its commercial availability and ease of purification from seed material.^65^ Glucoraphanin, however, is increasingly being used in such studies because of its importance to human health. The reduction of sulforaphane to erucin was first observed in a study with rats fed sulforaphane where erucin ITC conjugates were detected in bile and urine.^66^ More recently, the same type of transformation has been observed with both glucoraphanin and sulforaphane in a human fecal fermentation. During a batch fermentation of glucoraphanin with a human fecal inoculum^67^ a time‐dependent decrease in glucoraphanin concentration was observed with a corresponding increase in the levels of glucoerucin. This was also evident in the hydrolytic products of the fermentation where sulforaphane and sulforaphane nitrile accounted for less than 2% of the total products while erucin and erucin nitrile formed 28% and 67%, respectively. Thus a major change in the side chain structure occurred as well as the formation of nitriles as the dominant products which has implications for the bioactivity of glucoraphanin. Work with individual bacterial strains (Table 2) showed both glucoraphanin and glucoiberin to be converted to their corresponding reduced form to give glucoerucin and glucoiberverin while the hydrolytic products were the corresponding nitriles, that is, erucin nitrile and iberverin nitrile.^25^ Further work^23^ with other bacteria showed the same trend with the exception of *L. agilis* R16 and *E. casseliflavus* CP1 which could not metabolize glucoraphanin or glucoiberin while *E. coli* VL8 was able to biotransform both of these GSLs as well as others (Table 2). It was also observed in this study that both sulforaphane and sulforaphane nitrile were able to undergo this conversion to the reduced forms. Using a crude cell‐free protein extract of *E. coli* VL8, the reductase activity was shown to be NADPH‐ and Mg^2+^ ‐dependent. Given the importance of glucoraphanin in the diet, the oxidation‐reduction of the methylsulfinyl alkyl side chain requires more work particularly to see if glucoerucin or its corresponding ITC can be re‐oxidized in humans.

### 1.6 Bacterial Myrosinase Sequences and Mechanism of Activity

Almost all plant myrosinases belong to the GH1 family of β‐*O*‐glucosidases and are activated by ascorbate.^7^, ^68^, ^69^ Some insect myrosinases have also been characterized in particular from *Brevicoryne brassicae* which also belongs to the GH1 family of β‐*O*‐glucosidases.^70^, ^71^, ^72^ Most interrogation of the bacterial genomes for identification of myrosinase genes has been based on plant myrosinase gene sequences. This was the case for the recent work with *E. coli* 0157:H7 where mutations of the putative myrosinase genes were generated by gene replacement to confirm the identify.^30^ We have used a similar approach where the candidate genes from *E. casseliflavus* CP1 and *E. coli* VL8 were cloned based on sequences from the known genomes of *E. casseliflavus* NCCP‐53 and *E. coli* O83:H1 NRG 857C and overexpressed them in *E.coli* 44 although no myrosinase activity could be demonstrated.

Both plant and aphid myrosinases have been fully characterized.^68^, ^70^ Mechanistically the two enzymes are different with plant myrosinase utilizing a glutamic acid as a nucleophile with ascorbate^69^ acting as a catalytic base while aphid myrosinase functions as a typical β‐*O*‐glucosidase using two glutamate residues without a requirement for ascorbate.^72^ To date, ascorbate has only had a marginal effect on non‐plant myrosinases which is perhaps expected since plant myrosinase utilizes ascorbate as a cofactor in the active site as a base whereas most β‐O‐glucosidases use a glutamic acid residue.

**Figure 4:**
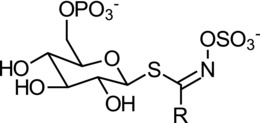
Generalized structure of a hypothetical 6‐P‐glucosinolate.

For bacterial myrosinases, little is known concerning the structure of myrosinase with the exception of *Citrobacter* Wye1 where a complete gene sequence has been identified.^34^ This sequence was based on the actual genome of *Citrobacter* Wye1 together with an N‐terminal sequence and peptide sequences from tryptic digests of the purified myrosinase. Unlike plant and aphid myrosinases, this enzyme belongs to the GH3 family of β‐*O*‐glucosidases (InterProt analysis^73^) and the full‐length myrosinase gene that encodes an N‐terminal signal peptide which presumably targets the protein to the periplasm. Other recent work has identified a 6‐phospho‐β‐glucosidase (*bglA, ascbB, chbF*) which was based on homology with plant myrosinase. Gene mutations were carried out and analyzed for their ability to metabolize sinigrin and it was found that the genes *bglA* and *ascbB* played an important role in sinigrin degradation by *E. coli* 0157:H7.^30^ It would be useful to express these genes and undertake detailed characterization to confirm their role as myrosinases. Interestingly, the *Citrobacter* Wye 1 myrosinase has strong homology (70%) with an *E. cloacae* β‐*O*‐glucosidase which is known to have myrosinase activity as well as high homology with other bacterial β‐*O*‐glucosidases. A feature of the GH3 β‐O‐glucosidase is the signature “SDW” conserved motif as is the case for *Citrobacter* Wye1 myrosinase and contains aspartate as the catalytic nucleophile rather than glutamate that is characteristic of GH1 plant myrosinases. There was very little homology between the *Citrobacter* Wye1 and plant or aphid myrosinases.^34^ If a 6‐phospho‐β‐glucosidase is responsible for the metabolism of sinigrin by *E. coli* 0157:H7 then it is possible that the GSL substrate requires phosphorylation at the 6‐hydroxyl position on the glucose residue of the GSL (**Figure** 4). ATP‐dependent β‐glucoside kinases are known and can phosphorylate a range of substrates such as the natural products salicin and amygdalin and artificial substrates such as iso‐propyl‐β‐D‐thioglucopyranoside.^74^ In a recent study using differential proteomics on *E. coli* VL8, a glucose‐specific phosphotransferase system was shown to be induced by sinigrin (in comparison to a control) which gives some evidence toward a phosphorylation step necessary for the hydrolysis of GSLs.^75^ Thus, a prerequisite phosphorylation of the glucose moiety might explain why it has not been possible to observe myrosinase activity in cell‐free protein extracts of some of the bacteria such as *L. agilis* R16 described in this review.

### 1.7 Diversity of Microorganisms Able to Metabolize Glucosinolates

Despite the limited number of studies on bacterial metabolism of GSLs, it is clear that this metabolic capacity is not limited to a single phylotype or a family of bacterial species. They include members of Firmicutes, Bacteriodetes, Actinomycetes, and Proteobacteria. There is good evidence to indicate that a high degree of horizontal gene transfer can occur between bacterial species in the environment^76^, ^77^ that may explain the observed diversity in bacterial groups able to metabolize the GSLs. The range of bacteria include both Gram‐positive and Gram‐negative, those that are rods or cocci, commensal, and those associated with pathogenic traits all have the capacity for GSL biotransformation. Also, the habitat of these bacteria is not limited to the GI tract although most of the studies are related to gut bacteria for their association with dietary GSLs. They are also found in soil and have been isolated from plant sources. No doubt, we will find many more relevant bacterial groups as we learn more about the gut bacteria and their metabolic capacity in both human and animal GI tract. In this review, we have focused only on bacterial metabolism but it is highly likely that in time we will discover other microbes, archaea, yeast, and fungi that are able to metabolize GSLs. Several studies have already reported the presence of myrosinase in *Aspergillus niger* 78, 79 and other fungi.^80^, ^81^

### 1.8 Conclusions and Future Work

In comparison to plants very little work has been carried out on the metabolism of GSLs by bacteria. Given the importance of GSLs in the human diet it has become desirable to investigate the mechanisms of their biotransformation in the gut, particularly with a view to increasing ITC production. This requires a much more detailed study to identify those bacteria that play a key role in ITC production as well as investigating why nitriles are often the end‐products. It may well be that ITCs are toxic to the bacteria that produce them and it then becomes preferable for nitrile production as a form of detoxification as is the case for some insects that metabolize GSLs.^82^ While specifier proteins that modify the outcome of GSL hydrolysis have been discovered in plants^36^ no such proteins have been found in bacteria although there is one case of an identified ETN in rat intestinal microbiota^38^ which suggests the presence of an ESP‐like protein although this requires confirmation. The role of sulfatases in GSL metabolism is still unclear although DS‐GSL metabolism to nitriles has been established and these enzymes require identification to confirm this role.

Human intervention studies have shown that there is a wide variation in the amount of ITC present in the urine and it is thought that this may reflect on differences in the composition of the microbiotica of individuals, that is, the ability of microbiota to generate ITCs.^83^, ^84^ These studies identified subjects that are low or high secretors of ITCs in their urine and indeed the fecal microbiota of high ITC secretors were more efficient at degrading glucoraphanin than those of low ITC secretors. However, using tRFLP, a relatively low resolution molecular profiling method, indicated that the gut bacterial communities are altered by consumption of cruciferous vegetables in all subjects. It was not possible however, to differentiate the composition of the gut microbiota between the two secretor groups.^83^, ^84^ Given that many of the identified metabolizers of the GSLs include groups of beneficial bacteria such as the lactobacilli and bifidobacteria that are often utilized as probiotics, this opens up potential opportunities to exploit such bacteria as dietary supplements with GSL‐containing foods that would provide health benefits particularly to low ITC secreting individuals as part of their personalized nutrition. Recently, a study has looked at the potential for expressing the glucotropaeolin biosynthetic pathway in *E. coli* together with a myrosinase of *B. brassicae* with some success as the authors were able to show the in vitro formation of benzylisothiocyanate.^85^ If this technology can be developed where ITCs are actually produced during fermentation then it could potentially allow the delivery of sulforaphane in the gut. However, such heterologous systems would be considered genetically modified organism (GMO) and would require regulatory approval.

Further characterization of bacterial myrosinases should be an important goal in understanding product formation from GSL hydrolysis. The sequences of bacterial myrosinase genes are likely to be different from plant and aphid myrosinases as was demonstrated by the recent characterization of the *Citrobacter* Wye1 enzyme.^34^ Once additional functional bacterial genes have been identified it will open up opportunities for genome mining of human and animal gut bacterial metagenomes which are becoming widely available as a result of many different microbiome sequencing projects. Approaches to identify myrosinase genes should include the determination of genomes together with peptide sequence analysis of partially or purified myrosinase. A synthesis of 6‐P‐GSL would enable the possibility of 6‐phospho‐β‐*O*‐glucosidases as myrosinases to be explored. *L. agilis* R16 produces large amounts of allylisothiocyanate from sinigrin yet does not inhibit its growth suggesting that the ITC cannot cross its cell wall or it has developed other resistance mechanisms. In contrast, *Citrobacter* Wye1 does not seem to produce allylisothiocyanate in vivo but to as yet an unknown molecule which might be a detoxification product or that the allylisothiocyanate is unstable. This may also be true of other bacteria and requires a more thorough study to determine potential detoxification mechanisms. For example, allylamine and benzylamine were obtained from sinigrin and glucotropaeolin, respectively in a human fecal fermentation.^47^ During our fermentation work, both with pure bacterial cultures and with mixed fecal bacteria we have never observed amines and it would be desirable to investigate further the occurrence of these compounds.

Of interest is the effect that ITC‐producing bacteria have on other microorganisms of the human gut. ITCs are known to have antibacterial properties^86^, ^87^, ^88^ and it is possible that there may be an overall negative effect on other functions of the gut microbiota. This however, would be dependent on the concentrations of ITCs in the human gut and as yet this question has not been fully addressed.^56^ A recent study has shown that the microbial conversion of GSLs to ITCs can be modified by the frequency of GSL consumption in rats and resulted in a change of the microbiota composition^89^ which is effectively an enrichment process for GSL‐utilizing bacteria. While some bacteria produce ITCs it is highly likely that other bacteria in the human gut will have the potential to detoxify these compounds so the situation with the microbiota is likely to be complex. This raises many questions on the importance and efficiency of gut bacteria in the generation of ITCs and competing detoxification processes and how this impacts on the bioavailability of ITCs.

## Conflict of Interest

The authors declare no conflicts of interest.

## References

[1] N. V. Matusheski , R. Swarup , J. A. Juvik , R. Mithen , M. Bennett , E. H. Jeffery , J. Agric. Food Chem. 2006, 54, 2069. 16536577 10.1021/jf0525277

[2] N. Fujioka , V. Fritz , P. Upadhyaya , F. Kassie , S. S. Hecht , Mol. Nutr. Food Res. 2016, 60, 1228. 26840393 10.1002/mnfr.201500889 PMC12262096

[3] A. Mazumder , A. Dwivedi , J. Du Plessis , Molecules 2016, 21, 416 10.3390/molecules21040416 27043505 PMC6273501

[4] P. Gupta , B. Kim , S. H. Kim , S. K. Srivastava , Mol. Nutr. Food Res. 2014, 58, 1685. 24510468 10.1002/mnfr.201300684 PMC4122603

[5] J. D. Hayes , M. O. Kelleher , I. M. Eggleston , Eur. J. Nutr. 2008, 47, 73. 18458837 10.1007/s00394-008-2009-8

[6] M. Traka , R. Mithen , Phytochem. Rev. 2009, 8, 269.

[7] A. M. Bones , J. T. Rossiter , Physiol. Plant 1996, 97, 194.

[8] M. J. Garson , J. S. Simpson , Nat. Prod. Rep. 2004, 21, 164. 15039841 10.1039/b302359c

[9] S. Patai , The Chemistry of Cyanates and Their Thio Derivatives 2010, 10.1002/9780470771532.ch6.

[10] K. K. Brown , M. B. Hampton , Biochim. Biophys. Acta ‐ Gen. Subj. 2011, 1810, 888. 10.1016/j.bbagen.2011.06.004 21704127

[11] G. R. Fenwick , R. K. Heaney , W. J. Mullin , Crit. Rev. Food Sci. Nutr. 1983, 18, 123. 6337782 10.1080/10408398209527361

[12] M. A. Greer , J. Am. Chem. Soc. 1956, 78, 1260.

[13] E. B. Astwood , M. A. Greer , M. G. Ettlinger , J. Biol. Chem. 1949, 181, 121. 15390398

[14] E. L. Oginsky , A. E. Stein , M. A. Greer , Exp. Biol. Med. 1965, 119, 360. 10.3181/00379727-119-30181 14328890

[15] L. Nugon‐Baudon , S. Rabot , J. M. Wal , O. Szylit , J. Sci. Food Agric. 1990, 52, 547.

[16] L. Nugon‐Baudon , O. Szylit , P. Raibaud , J. Sci. Food Agric. 1988, 43, 299.

[17] S. Rabot , L. Nugon‐Baudon , P. Raibaud , O. Szylit , Br. J. Nutr. 1993, 70, 323. 8399112 10.1079/bjn19930125

[18] S. Rabot , C. Guerin , L. Nugon‐Baudon , O. Szylit , in Proc. 9th Int. Rape‐Seed Congr. (Ed: GCIRC), Dorset Press, Dorchester 1995, p. 212.

[19] L. Elfoul , S. Rabot , N. Khelifa , A. Quinsac , A. Duguay , A. Rimbault , FEMS Microbiol. Lett. 2001, 197, 99. 11287153 10.1111/j.1574-6968.2001.tb10589.x

[20] R.‐H. Lai , M. J. Miller , E. Jeffery , Food Funct. 2010, 1, 161. 21776467 10.1039/c0fo00110d

[21] A. Brabban , C. Edwards , FEMS Microbiol. Lett. 1994, 119, 83. 8039675 10.1111/j.1574-6968.1994.tb06871.x

[22] M. Ll. Palop , J. P. Smiths , B. ten Brink , Int. J. Food Microbiol. 1995, 26, 219. 7577359 10.1016/0168-1605(95)00123-2

[23] V. Luang‐In , A. Narbad , C. Nueno‐Palop , R. Mithen , M. Bennett , J. T. Rossiter , Mol. Nutr. Food Res. 2014, 58, 875. 24170324 10.1002/mnfr.201300377

[24] V. Luang‐In , A. A. Albaser , C. Nueno‐Palop , M. H. Bennett , A. Narbad , J. T. Rossiter , Curr. Microbiol. 2016, 73, 442. 27301252 10.1007/s00284-016-1079-8

[25] J. A. Mullaney , W. J. Kelly , T. K. McGhie , J. Ansell , J. A. Heyes , J. Agric. Food Chem. 2013, 61, 3039. 23461529 10.1021/jf305442j

[26] R.‐H. Lai , M. J. Miller , E. H. Jeffery , FASEB J. 2009, 23, Supplement 561.4.

[27] D. L. Cheng , K. Hashimoto , Y. Uda , Food Chem. Toxicol. 2004, 42, 351. 14871576 10.1016/j.fct.2003.09.008

[28] F. B. Luciano , J. Belland , R. A. Holley , Int. J. Food Microbiol. 2011, 145, 69. 21146240 10.1016/j.ijfoodmicro.2010.11.028

[29] S. Herzallah , M. L. Lledó , R. Holley , J. Food Prot. 2011, 74, 2162. 22186059 10.4315/0362-028X.JFP-11-284

[30] R. P. Cordeiro , J. H. Doria , G. G. Zhanel , R. Sparling , R. A. Holley , Int. J. Food Microbiol. 2015, 205, 105. 25897994 10.1016/j.ijfoodmicro.2015.04.008

[31] B. Wassermann , D. Rybakova , C. Müller , G. Berg , Sci. Rep. 2017, 7, 17649. 29247170 10.1038/s41598-017-17949-z PMC5732279

[32] N. Tani , M. Ohtsuru , T. Hata , T. Naoki , T. Ani , Agr. Biol. Chem. 1974, 389, 1623.

[33] N. Tani , M. Ohtsuru , T. Hata , Agric. Biol. Chem. 1974, 38, 1617.

[34] A. Albaser , E. Kazana , M. H. Bennett , F. Cebeci , V. Luang‐In , P. D. Spanu , J. T. Rossiter , J. Agric. Food Chem. 2016, 64, 1520. 26820976 10.1021/acs.jafc.5b05381

[35] F. Cebeci , The Metabolism of Plant Glucosinolates by Gut Bacteria, PhD thesis, University of East Anglia, UK 2017.

[36] J. C. Kuchernig , M. Burow , U. Wittstock , BMC Evol. Biol. 2012, 12, 127. 22839361 10.1186/1471-2148-12-127 PMC3482593

[37] W. Brandt , A. Backenköhler , E. Schulze , A. Plock , T. Herberg , E. Roese , U. Wittstock , Plant Mol. Biol. 2014, 84, 173. 23999604 10.1007/s11103-013-0126-0

[38] M. Lu , K. Hashimoto , Y. Uda , Food Res. Int. 2011, 44, 1023.

[39] P. Gadler , K. Faber , Trends Biotechnol. 2007, 25, 83. 17150269 10.1016/j.tibtech.2006.11.006

[40] S. R. Hanson , M. D. Best , C. H. Wong , Angew. Chemie‐Int. Ed. 2004, 43, 5736. 10.1002/anie.200300632 15493058

[41] J. P. Wathelet , R. Iori , O. Leoni , P. Rollin , N. Mabon , M. Marlier , S. Palmieri , Biotechnol. Lett. 2001, 23, 443.

[42] A. J. MacLeod , J. T. Rossiter , Phytochemistry 1986, 25, 1047.

[43] F. L. Austin , C. Gent , I. Wolff , Can. J. Chem. 1968, 46, 1507.

[44] V. Luang‐In , Influence of Human Gut Microbiota on the Metabolic Fate of Glucosinolates, PhD thesis, Imperial College, UK 2013.

[45] J. Kopycki , J. Schmidt , S. Abel , C. D. Grubb , Biotechnol. Lett. 2011, 33, 1039. 21267762 10.1007/s10529-011-0530-y

[46] M. G. Ettlinger , A. J. Lundeen , J. Am. Chem. Soc. 1957, 79, 1764.

[47] B. Combourieu , L. Elfoul , A. M. Delort , S. Rabot , Drug Metab. Dispos. 2001, 29, 1440. 11602519

[48] K. Moeller , G. S. Nguyen , F. Hollmann , U. Hanefeld , Enzyme Microb. Technol. 2013, 52, 129. 23410922 10.1016/j.enzmictec.2012.12.003

[49] P. W. Ramteke , N. G. Maurice , B. Joseph , B. J. Wadher , Biotechnol. Appl. Biochem. 2013, 60, 459. 23826937 10.1002/bab.1139

[50] F. Carbonero , A. C. Benefiel , A. H. Alizadeh‐Ghamsari , H. R. Gaskins , Front. Physiol. 2012, November 3. 10.3389/fphys.2012.00448 PMC3508456 23226130

[51] V. Luang‐In , J. T. Rossiter , Songklanakarin J. Sci. Technol. 2015, 37, 625.

[52] J. R. Mays , R. L. W. Roska , S. Sarfaraz , H. Mukhtar , S. R. Rajski , Chem. Bio. Chem. 2008, 9, 729. 10.1002/cbic.200700586 18327862

[53] A. A. Al Janobi , R. F. Mithen , A. V. Gasper , P. N. Shaw , R. J. Middleton , C. A. Ortori , D. A. Barrett , J. Chromatogr. B Anal. Technol. Biomed. Life Sci. 2006, 844, 223. 10.1016/j.jchromb.2006.07.007 16931178

[54] D. B. Clarke , Anal. Methods 2010, 2, 310.

[55] D. Angelino , E. Jeffery , J. Funct. Foods 2014, 7, 67.

[56] D. Angelino , E. B. Dosz , J. Sun , J. L. Hoeflinger , M. L. Van Tassell , P. Chen , J. M. Harnly , M. J. Miller , E. H. Jeffery , Front. Plant Sci. 2015, 6, October 6. 10.3389/fpls.2015.00831 PMC4593958 26500669

[57] G. Barknowitz , W. Engst , S. Schmidt , M. Bernau , B. H. Monien , M. Kramer , S. Florian , H. Glatt , Chem. Res. Toxicol. 2014, 27, 188. 24422435 10.1021/tx400277w

[58] C. C. Conaway , D. Jiao , T. Kohri , L. Liebes , F. L. Chung , Drug Metab. Dispos. 1999, 27, 13. 9884304

[59] M. Bollard , S. Stribbling , S. Mitchell , J. Caldwell , Food Chem. Toxicol. 1997, 35, 933. 9463527 10.1016/s0278-6915(97)00103-8

[60] E. R. Brocker , M. H. Benn , J. Lüthy , A. von Däniken , Food Chem. Toxicol. 1984, 22, 227. 6538540 10.1016/0278-6915(84)90132-7

[61] J. Valette , B. Tiret , F. Boumezbeur , Anal. Biochem. 2017, 529, 216. 10.1016/j.ab.2016.08.003 27515993

[62] J. J. Morrison , N. P. Botting , Tetrahedron Lett. 2007, 48, 1891.

[63] S. Chevolleau , B. Joseph , P. Rollin , J. Tulliez , J. Labelled Comp. Radiopharm. 1993, 33, 671.

[64] J. T. Rossiter , J. A. Pickett , M. H. Bennett , A. M. Bones , G. Powell , J. Cobb , Phytochemistry 2007, 68, 1384. 17434192 10.1016/j.phytochem.2007.02.030

[65] W. Thies , FETT Wiss. Technol. Sci. Technol. 1988, 90, 311.

[66] K. Kassahun , M. Davis , P. Hu , B. Martin , T. Baillie , Chem. Res. Toxicol. 1997, 10, 1228. 9403174 10.1021/tx970080t

[67] S. Saha , W. Hollands , B. Teucher , P. W. Needs , A. Narbad , C. A. Ortori , D. A. Barrett , J. T. Rossiter , R. F. Mithen , P. A. Kroon , Mol. Nutr. Food Res. 2012, 56, 1906. 23109475 10.1002/mnfr.201200225

[68] W. P. Burmeister , S. Cottaz , H. Driguez , R. Iori , S. Palmieri , B. Henrissat , Structure 1997, 5, 663. 9195886 10.1016/s0969-2126(97)00221-9

[69] W. P. Burmeister , S. Cottaz , P. Rollin , A. Vasella , B. Henrissat , J. Biol. Chem. 2000, 275, 39385. 10978344 10.1074/jbc.M006796200

[70] H. Husebye , S. Arzt , W. P. Burmeister , F. V. Härtel , A. Brandt , J. T. Rossiter , A. M. Bones , Insect Biochem. Mol. Biol. 2005, 35, 1311. 16291087 10.1016/j.ibmb.2005.07.004

[71] A. M. E. Jones , M. Bridges , A. M. Bones , R. Cole , J. T. Rossiter , Insect Biochem. Mol. Biol. 2001, 31, 1. 11102829 10.1016/s0965-1748(00)00157-0

[72] A. M. E. Jones , P. Winge , A. M. Bones , R. Cole , J. T. Rossiter , Insect Biochem. Mol. Biol. 2002, 32, 275. 11804799 10.1016/s0965-1748(01)00088-1

[73] P. Jones , D. Binns , H. Y. Chang , M. Fraser , W. Li , C. McAnulla , H. McWilliam , J. Maslen , A. Mitchell , G. Nuka , S. Pesseat , A. F. Quinn , A. Sangrador‐Vegas , M. Scheremetjew , S. Y. Yong , R. Lopez , S. Hunter , Bioinformatics 2014, 30, 1236. 24451626 10.1093/bioinformatics/btu031 PMC3998142

[74] J. Thompson , F. W. Lichtenthaler , S. Peters , A. Pikis , J. Biol. Chem. 2002, 277, 34310. 12110692 10.1074/jbc.M206397200

[75] V. Luang‐In , A. Narbad , F. Cebeci , M. Bennett , J. T. Rossiter , Protein J. 2015, 34, 135. 25805049 10.1007/s10930-015-9607-0

[76] C. S. Smillie , M. B. Smith , J. Friedman , O. X. Cordero , L. A. David , E. J. Alm , Nature 2011, 480, 241. 22037308 10.1038/nature10571

[77] J. H. Hehemann , G. Correc , T. Barbeyron , W. Helbert , M. Czjzek , G. Michel , Nature 2010, 464, 908. 20376150 10.1038/nature08937

[78] M. Ohtsuru , I. Tsuruo , T. Hata , Agric. Biol. Chem. 1973, 37, 967.

[79] M. Ohtsuru , T. Hata , Agric. Biol. Chem. 1973, 37, 2543.

[80] J. P. Smits , W. Knol , J. Bol , Appl. Microbiol. Biotechnol. 1993, 38, 696.

[81] S. Galletti , E. Sala , O. Leoni , S. Cinti , C. Cerato , Soil Biol. Biochem. 2008, 40, 2170.

[82] V. Jeschke , J. Gershenzon , D. G. Vassão , Form. Struct. Act. Phytochem. 2015, 45, 163.

[83] F. Li , M. A. J. Hullar , Y. Schwarz , J. W. Lampe , J. Nutr. 2009, 139, 1685. 19640972 10.3945/jn.109.108191 PMC2728691

[84] F. Li , M. A. J. Hullar , S. A. A. Beresford , J. W. Lampe , Br. J. Nutr. 2011, 106, 408. 21342607 10.1017/S0007114511000274 PMC3137642

[85] F. Liu , H. Yang , L. Wang , B. Yu , ACS Synth. Biol. 2016, 5, 1557. 27389525 10.1021/acssynbio.6b00143

[86] A. C. Abreu , A. Borges , L. C. Simoes , M. J. Saavedra , M. Simoes , Med. Chem. 2013, 9, 756. 22974327 10.2174/1573406411309050016

[87] A. Borges , A. C. Abreu , C. Ferreira , M. J. Saavedra , L. C. Simões , M. Simões , J. Food Sci. Technol. 2015, 52, 4737. 26243895 10.1007/s13197-014-1533-1 PMC4519465

[88] A. Aires , V. R. Mota , M. J. Saavedra , E. A. S. Rosa , R. N. Bennett , J. Appl. Microbiol. 2009, 106, 2086. 19291240 10.1111/j.1365-2672.2009.04180.x

[89] X. Liu , Y. Wang , J. L. Hoeflinger , B. P. Neme , E. H. Jeffery , M. J. Miller , Nutrients 2017, 9, 262. 10.3390/nu9030262 PMC5372925 28287418

[90] C. Krul , C. Humblot , C. Philippe , M. Vermeulen , M. van Nuenen , R. Havenaar , S. Rabot , Carcinogenesis 2002, 23, 1009. 12082023 10.1093/carcin/23.6.1009

[91] J. Huber , G. Kranz , G. Kreibich , K. Beining , M. Kruger , F. Weissbach , Nahrung 1983, 27, 257. 6684210 10.1002/food.19830270319

[92] A. N. Olaima , B. Sobhi , R. A. Holley , J. Food Prot. 2014, 77, 2133. 25474062 10.4315/0362-028X.JFP-14-210

